# Exploring the therapeutic potential of autologous hematopoietic stem cell transplantation in progressive multiple sclerosis—a systematic review

**DOI:** 10.1111/ene.16427

**Published:** 2024-08-05

**Authors:** Bente Braun, Felix Fischbach, Lena Kristina Pfeffer, Johanna Richter, Dietlinde Janson, Nicolaus M. Kröger, Alice Mariottini, Christoph Heesen, Vivien Häußler

**Affiliations:** ^1^ Institute of Neuroimmunology and Multiple Sclerosis University Medical Centre Hamburg‐Eppendorf Hamburg Germany; ^2^ Department of Stem Cell Transplantation University Medical Center Hamburg‐Eppendorf Hamburg Germany; ^3^ Department of Neurosciences, Psychology, Drug Research and Child Health University of Florence Florence Italy; ^4^ Department Neurology II Careggi University Hospital Florence Italy; ^5^ Department of Neurology University Medical Centre Hamburg‐Eppendorf Hamburg Germany

**Keywords:** autologous hematopoietic stem cell transplantation, no evidence of disease activity, progression‐free survival, progressive multiple sclerosis, transplant‐related mortality

## Abstract

**Background and Purpose:**

The aim was to determine the value of autologous haematopoietic stem cell transplantation (aHSCT) as a therapeutic intervention for progressive multiple sclerosis (PMS) based on a systematic review of the current literature.

**Methods:**

All studies from the databases PubMed and Google Scholar published in English before February 2024 which provided individual data for PMS patients were systematically reviewed. PICO was defined as population (P), primary progressive MS and secondary progressive MS patients; intervention (I), treatment with aHSCT; comparison (C), none, disease‐modifying therapy treated/relapsing–remitting MS cohorts if available; outcome (O), transplant‐related mortality, progression‐free survival (PFS) and no evidence of disease activity.

**Results:**

A total of 15 studies met the criteria including 665 patients with PMS (74 primary progressive MS, 591 secondary progressive MS) and 801 patients with relapsing–remitting MS as controls. PFS data were available for 647 patients. PMS patients showed more severe disability at baseline than relapsing–remitting MS patients. The average transplant‐related mortality for PMS in 10 studies was 1.9%, with 10 deaths in 528 patients. PFS ranged from 0% to 78% in PMS groups 5 years after treatment initiation, demonstrating a high variability. No evidence of disease activity scores at 5 years ranged from 0% to 75%.

**Conclusion:**

Based on the available data, aHSCT does not halt progression in people with PMS. However, there appears to be evidence of improved outcome in selected patients. Due to the heterogeneity of the available data, more comprehensive clinical trials assessing the efficacy of aHSCT across different patient groups are urgently needed to reduce variability and improve patient stratification.

## INTRODUCTION

Multiple sclerosis (MS) is a chronic disease of the central nervous system (CNS) caused by neuroinflammation followed by neurodegeneration [1]. Phenotypically, 85%–90% of patients initially experience a relapsing–remitting form (RRMS) whilst 10%–15% of patients manifest with neurological deterioration without clinical relapses (primary progressive MS; PPMS) [2]. Within 20 years, 50% of RRMS patients develop secondary progressive MS (SPMS) [3].

Several disease‐modifying therapies (DMTs) with different mechanisms of action have been approved for treating RRMS [4].Whilst siponimod in SPMS and ocrelizumab in PPMS [5, 6] have shown some limited efficacy, there is no therapeutic efficacy of other DMTs in patients with progressive MS (PMS), partly due to the immunopathological shift towards a CNS intrinsic and compartmentalized smouldering neuroinflammation upheld by CNS‐residing immune cells in PMS [7]. Therefore, there is an urgent need for therapeutic approaches.

Autologous haematopoietic stem cell transplantation (aHSCT) has been used in the treatment of MS for almost 30 years [8] and is increasingly recognized as a treatment for aggressive, highly active MS. Several studies have indicated a superiority of aHSCT over DMTs in the treatment of MS [9, 10, 11, 12, 13, 14]. With its improved safety profile, aHSCT has been suggested as a potential first‐line therapy for people with MS [15]. In a recent meta‐analysis including 50 MS studies with a total of 4831 patients [16], a significant reduction in the Expanded Disability Status Scale (EDSS) score of 0.48 and a reduction of the annual relapse rate (ARR) was found after transplantation. The average progression‐free survival (PFS) at 5 years was 73%. Another study has described its ability to completely suppress detectable inflammatory activity [17]. Therefore, these results suggest that stem cell transplantation represents an effective therapy for active MS.

However, it remains controversial whether aHSCT may be considered as a sufficient treatment option for progressive MS [18]. Another meta‐analysis suggests that cohorts with a high proportion of PMS patients are associated with increased mortality and significantly higher progression rates [19]. Nevertheless, aHSCT has been used in PMS patients in the past due to the lack of alternative treatment options. Employing aHSCT as the last treatment option leads to already pronounced disability in the majority of progressive patients at the time of transplantation. Currently, there are only a limited number of studies that have analysed the outcomes of aHSCT in PMS patients in an ideal setting comparing aHSCT to other available treatment options. This review aims to summarize the data of aHSCT as a therapeutic intervention for PMS by systematically evaluating the available evidence.

## METHODS

### Search strategy

All studies published in English between 2006 and February 2024 were systematically collected. PubMed and Google Scholar were searched for eligible articles using the following keywords and Boolean operators: ‘multiple sclerosis’, ‘MS’, ‘progressive’ and ‘aHSCT’. Studies that were updated in subsequent trials were partially excluded from the analysis.

### Eligibility criteria

Trials were screened according to the PICO criteria, including population (P), studies consisting only of PMS patients or providing individual outcomes for PMS patients only; intervention (I), treatment with aHSCT with either reduced, intermediate or high conditioning regimen; comparison (C), comparison with an aHSCT‐treated RRMS cohort or a DMT‐treated PMS cohort is desirable but its absence is not an exclusion criterion; outcome (O), transplant‐related mortality (TRM), PFS and no evidence of disease activity (NEDA‐3).

### Endpoints

Progression‐free survival was defined as survival in the absence of worsening of neurological symptoms with an EDSS increase of >1 for baseline EDSS ≤5 or an increase of >0.5 for baseline EDSS >5. NEDA‐3 was defined as the absence of relapses, magnetic resonance imaging (MRI) activity and no EDSS progression as previously defined. TRM was defined as mortality within the first 100 days post‐transplantation.

### Data extraction

Data extraction was performed and controlled independently by two investigators. For each study, the year of publication, country of origin, sample size, follow‐up period, type of MS, age and EDSS at baseline, disease duration, number of previous treatments, previous ARR, conditioning regimen and sex were extracted. Follow‐up information (PFS, TRM, NEDA‐3, number of relapses) was also collected if available. A total of 15 studies met the criteria including 665 patients with progressive MS (74 PPMS, 591 SPMS). PFS was available for 647 patients. In two studies, a direct comparison with a similar cohort treated with DMTs was available. Most studies clearly defined selection criteria for disease courses, whereas others did not [20, 21, 22, 23].

### Statistical analysis

Analysis was conducted using R 4.2.2 software. A pooled estimate for the TRM was calculated by dividing the number of deaths after aHSCT by the total number of transplanted patients. If the exact values for PFS or NEDA‐3 were not given in the text, they were extracted graphically from the figures (Kaplan–Meier survival curves). The studies by Boffa et al. [25], Mariottini et al. [27] and Su et al. [33] were partially excluded from the analysis as they were updated in subsequent studies. Nevertheless, false duplication of patient numbers cannot be excluded.

## RESULTS

### Baseline characteristics

Baseline characteristics of patients with PPMS, SPMS and RRMS are summarized in Table 1. The mean follow‐up time ranged from 1.6 to 9.6 years. At treatment initiation, PMS patients were up to 5 years older than those with RRMS. This age difference is particularly evident in Burt et al. [26] or Mancardi et al. [22] with an age at the initiation of therapy of 40.0 and 37.9 years in PMS patients and 35.9 and 33.0 years in RRMS patients. Additionally, baseline EDSS scores were higher in the PMS cohorts compared to the RRMS cohorts at the time of transplantation. Twelve out of 15 studies reported a baseline EDSS score of at least 6.0 in the PMS cohorts. The PMS cohorts had a mean disease duration ranging from 2.5 to 13.7 years prior to transplantation. The patients mostly underwent intermediate‐intensity conditioning, which involved carmustine (BCNU), etoposide, cytarabine and melphalan (BEAM) with or without anti‐thymocyte globulin (ATG) and in one study cyclophosphamide (Cy). The other cohorts received both low‐, intermediate‐ and high‐intensity conditioning regimens containing ‘mini BEAM’ (BCNU, melphalan), rituximab or busulfan, amongst others. As reported in the literature, approximately two‐thirds of the patients were female in analogy to the epidemiological properties of MS.

**TABLE 1:** Baseline characteristics.

Studies	Country	Disease course (*n*)			Follow‐up median (range)			Mean age		Mean EDSS base		Mean DD (SD)		No. of previous treatments (SD)		Previous ARR		Conditioning regimen	Sex (F) *n* (%)		Year of transplant
		RRMS	PPMS	SPMS	Total	DMT	PMS	DMT	PMS	DMT	PMS	DTM	PMS	DMT	PMS	DMT	PMS		DMT	PMS	
Boffa et al. [24]	Italy	217		69		3.1 (1.7–6.4)	6.8 (3.2–11.8)	37.8 (7.2)	38.1 (7.7)	6.3 (0.8)	6.2 (0.9)	13.7 (6.1)	13.7 (6.5)	2.3 (1.4)	2.4 (1.2)	0.90 (1.02)	1.08 (1.12)	Intermediate mix	131 (60.1)	45 (65.2)	1997–2019
Mariottini et al. [12]	Italy	0		31		7.6 mean	8.2 mean	**42.8 (7.09)**	**39.3 (7.27)**	5.7 (1.01)	5.9 (0.87)	13.8 (6.73)	13.7 (5.28)	1.3 (0.99)	3.0 (1.30)	0.46 (0.44)	0.56 (0.63)	BEAM + ATG	38 (61)	23 (74)	1991–2018

Studies	Country	Disease course (*n*)			Follow‐up median (range)			Mean age		Mean EDSS base		Mean DD (SD)		No. of previous treatments (SD)		Previous ARR		Conditioning regimen	Sex (F) *n* (%)		Year of transplant
		RRMS	PPMS	SPMS	Total	RRMS	PMS	RRMS	PMS	RRMS	PMS	RRMS	PMS	RRMS	PMS	RRMS	PMS		RRMS	PMS	
Boffa et al. [25]	Italy	122	2	86	6.2				34.8		6							Mixed			1997–2019
Burt et al. [26]	USA	414	0	93	2.7			35.9 (7.9)	40 (6,9)	3.9 (1.4)	5.2 (1.3)	6.3 (4.6)	11 (4.6)	3.6 (1.43)	4.15 (1.68)			Mixed	262 (63.3)	54 (58)	2003–2019
Mariottini et al. [27]	Italy	26	0	26			8.2 (2.3–18.5)		37 (27–58) median		6.0 (4.0–7.5) median		9 (4–25) median		3 (1–5) median		0.8 median (0.63–1.03)	BEAM + ATG		19 (73)	1999–2016
Nicholas et al. [20]	UK	58	22	40	1.6 (0.5–7.1).			40.2 (8.7)	43.6 (8.4)	6.0 (4.0–6.0)	6.5 (6.0–6.5)	9.2 ± 6.0	9.8 (4.5)	2.3 (1.3)	1.6 (1.1)	0.71	0.36	Cy‐ATG (2 exceptions)	33 (57)	19 (47)	2012–2019
Moore et al. [28]	Australia	20	0	15	3 (1–5.5)						Total 6 median							BEAM + ATG			2010–2016
Casanova et al. [29]	Spain	28	0	10	8.4	5.9	9.6	36.4 (9.1)	37.5 (9.1)	5.0 (1.3)	6.0 (0.7)	10.0 (8.5)	8.0 (4.4)	3 (1–6)	2 (1–4)	0.89 (0.9)	0.8 (1.1)	BEAM + ATG	20 (71.4)	7 (70.0)	1999–2015
Muraro et al. [21]	Italy	46	32	186	6.6						6,5							Mixed			1995–2006
Shevchenko et al. [30]	Russia	43	18	35	4.1			32.7 (18–51)	35.9 (18–54)	1.5 (1.5–4.5) median	5.0 (2.0–8.5) median	4 (0.5–10)	9.5 (2.5–24)					Low intensity	26 (60)	23 (43)	2005–2011
Mancardi et al. [22]	Italy	33	0	41	4	3.4 (0.8–9.6)	4.9 (0.2–9.7)	**33.0 (16–52)**	**37.9 (26–53)**	6.3 (3.5–9)	6.3 (4.5–7.5)	**8.3 (1–18)**	**13 (5 4–28)**			**2.8 (0–8)**	**1.1 (0–4)**	BEAM + ATG		/	1996–2008
Krasulová et al. [23]	Czechia	11	0	15	5.5	1.6 (0.92–11)	8 (2.5–10.8)	27 (19–37)	35 (20–44)	4.5 (2.5–7.0)	6.5 (6.0–7.5)	4 (2–9)	9 (5–19)			2 (1–5)		BEAM + ATG	6 (54)	9 (60)	1998–2008
Xu et al. [31]	China	0	0	37			4.1 (3.0)		35 (8.5)		6.6 (1.2)		6.0 (5.5)					Modified BEAM			
Ni et al. [32]	China	0	2	19			3.5 (0.5–5.4)		37 (15–58)		7.5 (5.0–9.5)		2.5 (0.5–8)					Cy/BEAM			
Su et al. [33]	China	0	0	15			2.9 (0.8–4.1)		36 (20–51)		6.0 (4.5–7.5)		3 (1.3–13)					Modified BEAM			2001–2005

Studies	Country	Disease course (*n*)			Follow‐up median (range)			Mean age		Mean EDSS base		Mean DD (SD)		No. of previous treatments (SD)		Previous ARR		Conditioning regimen	Sex (F) *n* (%)		Year of transplant
		RRMS	PPMS	SPMS	Total	RRMS	PPMS	RRMS	PPMS	RRMS	PPMS	RRMS	PPMS	RRMS	PPMS	RRMS	PPMS		RRMS	SPMS	
Nicholas et al. [20]	UK	58	22	40	1.6 (0.5–7.1)				45.8 (8.7)		6.0 (4.9–6.5)		6.6 (4.1)		0.14 (0.3)		0.05	Cy‐ATG (2 exceptions)		6 (27)	2012–2019

*Note*: The baseline characteristics of the 15 studies are displayed. The PMS cohorts listed in rows one and two are compared with a cohort treated with DMTs, whilst the cohort in the last row only includes PPMS patients and is therefore listed separately. Bold figures indicate a significant difference between the RRMS and PMS cohorts. Numbers given in months have been converted to years where necessary to simplify comparison.

Abbreviations: ARR, annual relapse rate; ATG, anti‐thymocyte globulin; BEAM, carmustine, etoposide, cytarabine and melphalan; Cy, cyclophosphamide; DD, disease duration; DMT, disease‐modifying therapy; EDSS, Expanded Disability Status Scale; PMS, progressive multiple sclerosis; PPMS, primary progressive multiple sclerosis; RRMS, relapsing–remitting multiple sclerosis; SPMS, secondary progressive multiple sclerosis.

### Transplant‐related mortality in PMS

The pooled TRM for PMS cohorts was 1.9%, with 10 deaths in 531 patients across the 10 studies that reported mortality separately for the PMS cohort. Three studies were excluded as subsequent studies exist and two studies did not provide the TRM exclusively for PMS patients but for the entire cohort. Therefore, these studies were not included in the calculation of the TRM for PMS patients.

**FIGURE 1:**
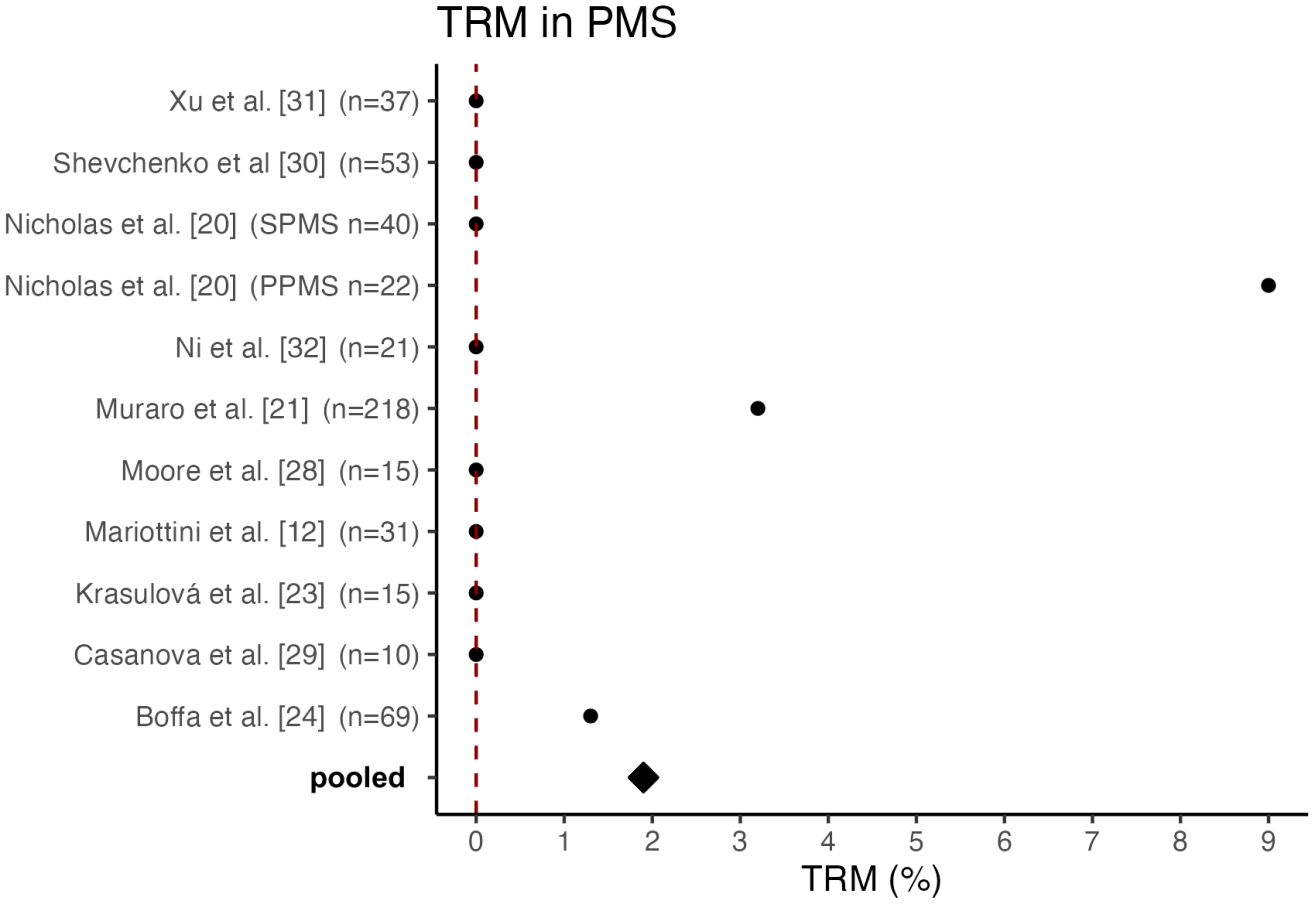
Transplant‐related mortality in PMS. Depicted are the TRM rates in PMS for each of the analysed studies, along with the resulting pooled value. Two studies did not report a TRM for PMS patients and three studies were excluded as subsequent studies exist. The points represent the TRM of the associated study, and the square represents the pooled TRM. The numbers in parentheses indicate the sample size of PPMS and SPMS patients in each cohort. PMS, progressive multiple sclerosis; PPMS, primary progressive multiple sclerosis; SPMS, secondary progressive multiple sclerosis; TRM, transplant‐related mortality.

Boffa et al. [24] reported a TRM of 1.3% attributed to the death of one patient 56 days after transplantation due to intracranial haemorrhage following treatment for a pulmonary artery embolism. In Muraro et al. [21], eight patients (2.8%) died within the first 100 days after transplantation, including seven people suffering from PMS. However, in most studies the TRM rate was 0% in the cohorts with PMS [12, 23, 27, 28, 29, 30, 31, 33]. Nicholas et al. [20] reported a TRM of 0% in their SPMS cohort; however, in the PPMS cohort two out of 22 patients died, resulting in a TRM of 9% for people with PPMS [20]. An overview of the TRM of the studies analysed is provided in Figure 1.

### Progression‐ and relapse‐free survival

**FIGURE 2:**
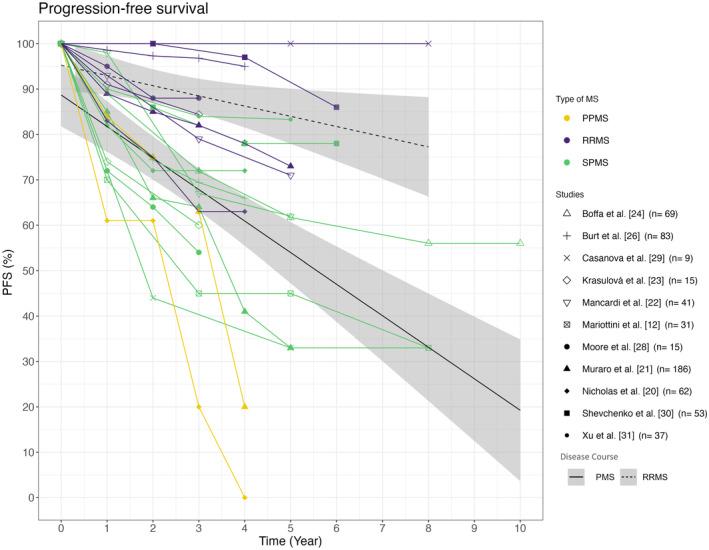
Overview of the PFS in PMS compared to RRMS. PFS rates of individual studies segmented by the MS disease courses are depicted. Time is measured in years after therapy initiation. Purple lines represent RRMS, green lines symbolize SPMS and yellow lines PPMS. The symbols indicate the corresponding study. A solid black line depicts the PFS average for PMS cohorts, whilst a dotted line represents RRMS cohorts. The numbers in parentheses indicate the sample size of PPMS and SPMS patients in each cohort. PFS, progression free survival; PMS, progressive multiple sclerosis; PPMS, primary progressive multiple sclerosis; RRMS, relapsing–remitting multiple sclerosis; SPMS, secondary progressive multiple sclerosis.

Autologous HSCT does not appear to achieve the same level of efficacy in PMS patients as in RRMS patients over a period of up to 10 years. Four years after treatment initiation, PFS ranged from 0% to 78% in the PMS groups. In cohorts consisting only of RRMS patients, the PFS ranged from 63% to 100% 4 years after transplantation, therefore indicating a higher PFS rate compared to PMS cohorts. Overall, the studies demonstrate varying results regarding PFS. Specifically, the three studies including long‐term data with up to 8–10 years of follow‐up reported divergent results with a PFS ranging from 57.2% to 30% after 10 years [25, 27, 29]. An overview of the PFS of the studies listed in Table 1 is provided in Figure 2.

In 2022, Mariottini et al. [12] investigated the use of aHSCT in PMS compared to low‐dose Cy in a non‐randomized setup. The study included 31 stem cell transplant patients matched 2:1 with a control group of 62 patients. The PFS of the aHSCT cohort and the Cy cohort did not differ significantly, with rates of 45% and 33% as well as 48% and 30% at 5 and 8 years, respectively. However, there was a significant difference in relapse‐free survival in favour of aHSCT, despite similar baseline ARR scores. In the aHSCT cohort, no relapse occurred after 5 years (100% at any time), whilst the relapse‐free survival in the control group was 71% at 2 years and 52% at 5 years [12]. Other studies have also reported no new relapses in PMS after aHSCT [12, 20]. Additionally, Burt et al. [26] reported a relapse‐free survival of 98.1% at 5 years.

A recent study [24] reported the outcomes of propensity‐score‐matched treatment groups. Sixty‐nine SPMS patients who received aHSCT were compared to 217 patients treated with anti‐inflammatory DMTs (beta interferons, azathioprine, glatiramer acetate, mitoxantrone, fingolimod, natalizumab, methotrexate, teriflunomide, cyclophosphamide, dimethyl fumarate and alemtuzumab). The study found that PFS was maintained in a significantly higher proportion of patients treated with aHSCT at 61.7% after 5 years compared to 46.3% in the DMT cohort. Further the aHSCT cohort showed a significant reduction of the ARR to 0.02 compared to 0.45 in the DMT‐treated cohort.

### Expanded Disability Status Scale improvement

Some studies have reported an improvement of the EDSS score after aHSCT, although the effect persisted partly only temporarily. The cohort published by Burt et al. in 2021 [26] reported the course of EDSS scores of 93 SPMS patients who converted to SPMS within the last 2 years. Amongst 35 patients with active SPMS, defined by at least one gadolinium‐enhancing lesion observed 1 year prior to transplantation, a significant decrease of the EDSS score was observed 3 years post transplantation compared to patients without active disease course [26]. Boffa et al. reported a significantly higher proportion of patients with a confirmed EDSS improvement of 38.8% compared to 7.8% in the DMT cohort after 3 years. Furthermore stable EDSS scores were found after aHSCT [24]. In a multicentre analysis conducted by Muraro et al. a significantly higher decrease in the EDSS score was observed in the RRMS cohort within 1 year of transplantation compared to PMS patients (RRMS −0.76, PMS −0.14). A short‐term improvement was also observed in the PMS cohort, albeit only temporary [21].

### No evidence of disease activity 3

**FIGURE 3:**
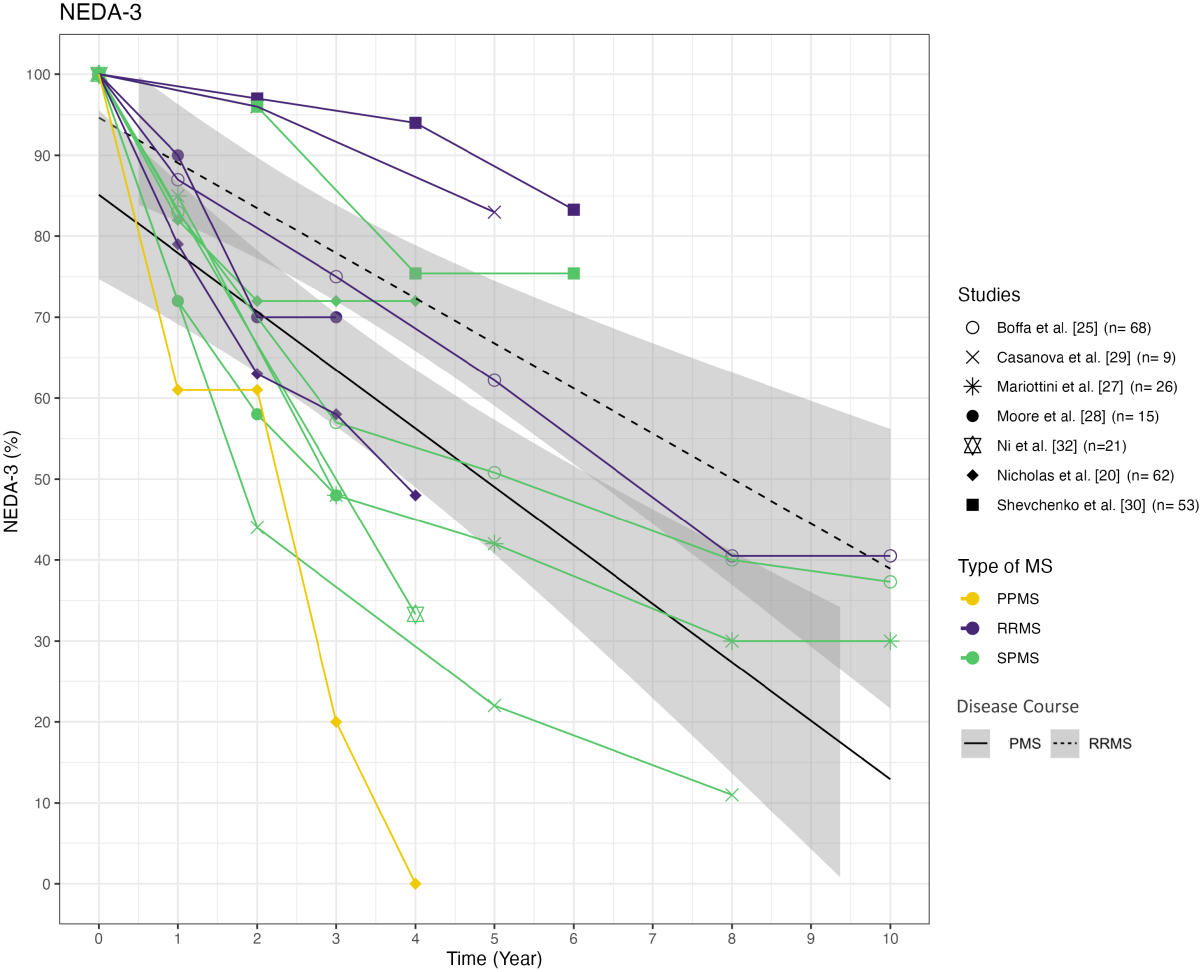
NEDA‐3 in PPMS, SPMS and RRMS cohorts. The NEDA‐3 rates reported in the analysed studies, categorized by the MS disease course, are depicted. Time is measured in years after therapy initiation. The purple lines represent RRMS, green lines symbolize SPMS and the yellow line symbolizes PPMS. The symbols indicate the corresponding study. The numbers in parentheses indicate the sample size of PMS patients in each cohort. NEDA, no evidence of disease activity; PMS, progressive multiple sclerosis; PPMS, primary progressive multiple sclerosis; RRMS, relapsing–remitting multiple sclerosis; SPMS, secondary progressive multiple sclerosis.

Seven studies reported NEDA‐3 results, as shown in Figure 3. Overall, NEDA‐3 scores ranged from 22% to 75% for cohorts with SPMS 5 years after transplantation. Particularly high NEDA‐3 scores were achieved 4 years after transplantation in the SPMS cohorts of Nicholas et al. (*n* = 40) and Shevchenko et al. (*n* = 35), with NEDA‐3 scores of 72% and 75%, respectively [30]. It is noteworthy that the SPMS cohort of Nicholas et al. achieved a higher NEDA‐3 rate than their RRMS cohort (*n* = 58), mostly reflecting a higher proportion of new focal inflammatory events (relapses and/or MRI activity) inducing NEDA failure in the latter [20].

In contrast, Boffa et al. [25], Mariottini et al. [27] and Casanova et al. [29] report lower NEDA‐3 rates of 50.8%, 42.0% and 22.0%, respectively, after 5 years. The lowest NEDA‐3 rates were found in the PPMS cohort by Nicholas et al. [20].

Mariottini et al. [12] reported only the NEDA‐2 outcome in their analyses, defined as the absence of relapses and EDSS progression, which is why the results are not shown in Figure 3. The analysis showed no significant difference in the NEDA‐2 rates of 45% and 36% for the aHSCT and Cy groups respectively after 5 years [12].

## DISCUSSION

Autologous HSCT is an important treatment for active MS and is increasingly recognized as a treatment option for patients with PMS. However, the results of available analysis are controversial.

### Reasonable safety profile of aHSCT in recently transplanted PMS patients

When evaluating the safety profile of aHSCT in autoimmune diseases rather than in haematological indications, it is important to consider that MS is a less life‐threatening disease. Therefore, potentially increased therapy‐related mortality demands more careful evaluation. With reduced life expectancy in disabled MS patients compared to the general population [34] treatment‐related mortality needs to be assessed in PMS compared to RRMS. Further, it is important to consider potential adverse effects such as opportunistic infections, secondary immunodeficiencies, infertility and secondary malignancies [13, 35]. The pooled mortality of the 10 available studies was 1.9%. The majority of the 15 available studies reported a TRM rate of 0%; in others very few patients died within the first 100 days after transplantation. Notably, the study with the highest absolute number of transplant‐related deaths referred to aHSCT performed between 1995 and 2006 and thereby represents one of the earliest cohorts, which potentially negatively impacts the pooled TRM rate due to improved transplant care and patient selection in more recent studies.

Nicholas et al. identified a relatively high TRM of 9% in PPMS patients (two out of 22 patients). The deceased patients both showed a high baseline EDSS score of 6.5, were aged 42 and 58 years, and had minor comorbidities [20]. According to an older meta‐analysis, a higher TRM is significantly associated with both a high baseline EDSS score and a progressive disease course [19]. Thus, these factors partly explain the high TRM in the study mentioned. However, it should be noted that the higher TRM observed in PMS cohorts could also be associated with comorbidities, such as an increased risk of infection and physical impairment.

The meta‐analysis conducted by Sormani et al. revealed a higher TRM of 3.4% in cohorts with a greater proportion of SPMS patients, compared to a TRM of 1.0% for relapsing courses, which may be partly due to the inclusion of older studies with small sample sizes. Only three out of the 15 included studies conducted all transplants after 2005. Out of 764 patients in total, Sormani et al. observed only one transplant‐related death after 2005, presumably due to the increasing experience, improved patient selection and the use of less toxic conditioning regimens. No significant associations were found regarding the conditioning regimens. Patients who underwent a low‐intensity regimen had a TRM of 0%, however [19]. In comparison, a more recent subgroup analysis found a TRM of 0% in the PPMS and SPMS subgroups and 1% in relapsing–remitting courses, which contradicts previous meta‐analyses [36]. However, the study with the highest absolute number of transplantation‐related deaths was not included. In this study, Muraro et al. [21] analysed the results of 25 centres involving 281 patients (46 RRMS, 216 PMS) with a median follow‐up of 6.6 years. In the entire cohort, eight patients died within 100 days after transplantation. Of these, seven patients had a progressive disease course, resulting in a TRM of 3.2% for PMS patients compared to 2.2% for RRMS patients. These high TRM rates may be attributed to the fact that aHSCT was conducted between 1995 and 2006; since then transplant‐related deaths have become rare. Throughout the observation period, PMS and high‐intensity conditioning regimens were associated with an increased incidence of mortality, with high baseline EDSS remaining the only significant predictor in a multivariate analysis [21]. The meta‐analysis conducted by Nabizadeh et al. [16] included 50 studies with 4831 patients and found a relatively high mortality rate of 4.0% regardless of the course of the disease. Unlike other studies, the TRM in this analysis did not refer to the period of 100 days after transplantation but to a period up to 5 years after transplantation. Due to the differing definition of TRM, data cannot be easily compared with those of other studies [16]. Despite the improved mortality rates after aHSCT in recent studies, it is important to note that the overall TRM seems to be higher in PMS than in RRMS cohorts, presumably due to comorbidities and a less favourable baseline.

Finally, with regard to the safety profile, it should be emphasized that a thorough risk–benefit analysis should be carried out for every patient. This is particularly important in the context of progressive disease, where treatment effects are less clear, as it is challenging to determine the degree of still ongoing inflammation in the CNS. Even the absence of classical signs of high‐grade focal inflammatory activity such as relapses or new MRI lesions does not exclude compartmentalized smouldering inflammation by resident immune cells [37], which could possibly be targeted by conditioning chemotherapy. Therefore, further investigation of aHSCT in multiple sclerosis is urgently needed to provide evidence for optimal patient selection, considering not only efficacy but also safety profile.

### Autologous HSCT demonstrates variable impact on PFS in PMS cohorts

Variations in outcomes of PFS were also observed amongst the other studies. It appears that aHSCT has a positive impact on the PFS in certain PMS cohorts, although not with the same efficacy as in patients with RRMS. Considering that aHSCT represents a potent form of immunomodulation, the improved response in disease courses characterized by high levels of inflammatory activity is plausible. A direct effect of the therapy on the ongoing neurodegenerative processes is less likely and the positive effect of aHSCT on PFS in PMS may be based on the suppression of inflammatory processes further driving neuronal loss. The wide variation in PFS observed across the individual studies may also be attributed to differing proportions of patients with ongoing inflammation within the progressive group. Despite the presence of PMS, these patients may still experience relapses or MRI activity. Supporting this hypothesis, relapses in the year prior to aHSCT were associated with higher PFS in PMS patients in the study by Boffa et al.; no effect of the intensity of the conditioning regimen was observed [25]. Further, Burt et al. observed an EDSS improvement in their active SPMS cohort in contrast to their non‐active SPMS [26]. Moreover, the choice of conditioning regimen not only affects the TRM but also influences the therapeutic response due to varying effectiveness. Future studies should specifically focus on investigating this effect in a structured manner, particularly in progressive patients.

The three cohorts reporting the highest PFS rates in PMS patients were amongst the youngest with a mean age at baseline of 34.8, 35.9 and 35.0 years [25, 30, 31]. An average PFS was observed in the analysed studies of approximately 61% for PMS and 86% for RRMS after 4 years. It is important to note that the sample size of the studies varies, which may cause distortions in the regression line. Similar results were found in the meta‐analysis published by Zhang and Liu in 2020 [36]. The 1626 MS patients included in the study showed a PFS of 81% for RRMS, 78% for PPMS and 60% for SPMS. However, the high PFS proportion of 78% in the PPMS cohort in this study is surprising [36]. The high rate of PFS calculated for the PPMS cohort may be attributed to the inclusion of only two studies, one of which has a small sample size and the other evaluates a self‐reported EDSS score with a median follow‐up of 1 year.

According to Boffa et al. [24], aHSCT may be superior to class 3 medications in terms of PFS in PMS. PMS patients treated with aHSCT were more likely to experience a sustained disability improvement [24]. The two available studies that compared aHSCT to DMTs in PMS patients [12, 24] presented different PFS rates for the PMS cohorts after transplantation. Mariottini et al. found similar PFS rates in their Cy cohort compared to their aHSCT cohort, possibly due to some effectiveness of both treatments. The two cohorts did not differ in terms of their baseline characteristics except for the stem cell patients being younger. To guarantee an objective comparison of PFS in all analysed studies, the differing definitions of progression in the two cohorts of Mariottini have been equalized [12, 27]. According to the meta‐analysis conducted by Sormani et al., disease course was the only significant factor associated with the 2‐year progression rate. Baseline EDSS values, conditioning regimes and patient age at transplantation did not show any significant association with the progression rate. Muraro et al. also found that RRMS was associated with higher rates of PFS, although there were no significant differences between PPMS and SPMS. In contrast to Sormani et al., a younger age and less than three previous DMTs were also independently associated with a higher PFS in the analysis [21].

Furthermore, the therapeutic effect on disease progression may evolve with a certain delay, since aHSCT primarily targets acute inflammation, assuming that degeneration might be secondary to inflammation, which is anything but clear. Consequently, progressive patients might show a continued EDSS worsening within the first months after transplantation, followed by a therapy‐induced plateau or at least deceleration in EDSS progression. This scenario would be classified as treatment failures in a traditional PFS analysis, thereby underestimating the therapeutic potential of aHSCT. Such a temporal stratification of PFS analyses in terms of a re‐baselining could thus be more sensitive but is currently not feasible due to limited availability of the data.

### Limited sustained effect of aHSCT in PMS on NEDA‐3 rates

To assess the efficacy of aHSCT in more detail, NEDA‐3 rates of the studies were analysed. Comparing Figures 2 and 3, it seems that NEDA‐3 failure was mostly driven by EDSS progression, but NEDA‐3 rates are only available in a limited number of studies. Particularly high NEDA‐3 rates were attained in the SPMS cohorts of Nicholas et al. (*n* = 40) and Shevchenko et al. (*n* = 35) after 4 years with 72% and 75%, respectively. In Shevchenko et al., high NEDA‐3 rates may be due to the low baseline EDSS value of 5.0, which is the lowest amongst the 15 cohorts. Furthermore, the patients were relatively young at treatment initiation with an average age of 35.9 years. Patients were conditioned using a low‐intensity protocol [30]. In contrast, patients in the Nicholas et al.'s study were amongst the oldest and most disabled compared to the other 14 studies. Despite the less favourable baseline characteristics, the high NEDA‐3 rates may be attributed to the patients' high disease activity prior to transplantation and by this stronger presence of inflammation [24]. Within the year prior to transplantation, 91% of the SPMS patients developed at least one new T2 lesion, 53% exhibited gadolinium‐enhancing lesions and the ARR of 0.36 was also quite high for SPMS. The short average observation period of 21 months may also have biased the results [20].

The lowest NEDA‐3 rates in PMS were found in the study conducted by Casanova et al. with 22% and 11% after 5 and 8 years, respectively. These rates could be attributed to the small sample size (*n* = 10) even though the disease duration and baseline EDSS were amongst the most favourable of the studies and the cohort showed a high proportion of relapsed patients (44%) with relapse‐free survival similar to the RRMS cohort. However, their PMS cohort showed the greatest difference to their transplanted RRMS cohort with 83% compared to 22% in PMS patients 5 years after transplantation [29].

Out of the 15 trials, only 74 of the 665 patients exhibited a PPMS course. Only two trials [20, 21] reported PFS rates for PPMS patients separately with discouraging results. Although the data might indicate that aHSCT is more effective in SPMS than in PPMS, the small sample size limits the ability to assess effectiveness in PPMS. Consequently, both the PFS rate and the NEDA‐3 results provide limited evidence for the efficacy of aHSCT in PPMS.

### Conclusion

Overall, the available studies do not allow strong conclusions, making it challenging to assess the effectiveness of aHSCT in PMS patients. This may be partly due to the different baseline characteristics of the cohorts and the limited number of studies, most of which provided small sample sizes. However, patients with an active disease course exhibit symptom stability, albeit often only temporarily.

Based on this analysis, patient selection considering disease activity and appropriate baseline characteristics seems to be important to achieve desired outcomes. aHSCT seems to have a particularly large effect in patients who are younger than 45 years of age, are less impaired (EDSS ≤6) and show acute disease activity and should therefore be considered for aHSCT. Based on the available data, it appears that PPMS patients benefit less than SPMS patients. Existing studies indicate a potential superiority of aHSCT over DMTs in PMS; however, randomized controlled trials to validate the efficacy and safety of aHSCT in PMS are necessary.

### Limitations

This work has relevant limitations. First, there could be a potential publication bias caused by the inclusion of published trials only. Further, it cannot be excluded that some patients have been double counted as many studies were conducted in the same country. Secondly, the published trials were mostly case series and retrospective database studies, and some studies were excluded because no subgroup analysis was available. Thirdly, there was a great heterogeneity between the groups in terms of baseline characteristics and follow‐up time. Due to the small sample size and our hypothesis, it was decided to summarize and interpret the available data in a systematic review.

## AUTHOR CONTRIBUTIONS

**Bente Braun:** Writing – original draft; visualization; formal analysis. **Felix Fischbach:** Writing – review and editing; formal analysis. **Lena Kristina Pfeffer:** Writing – review and editing. **Johanna Richter:** Writing – review and editing. **Dietlinde Janson:** Writing – review and editing. **Nicolaus M. Kröger:** Writing – review and editing. **Alice Mariottini:** Writing – review and editing. **Christoph Heesen:** Conceptualization; writing – review and editing. **Vivien Häußler:** Conceptualization; writing – review and editing; visualization.

## FUNDING INFORMATION

This research did not receive any specific grant from funding agencies in the public, commercial or not‐for‐profit sectors.

## CONFLICT OF INTEREST STATEMENT

None.

## Data Availability

The data that support the findings of this study are available from the corresponding author upon reasonable request.
